# Intraoperative molecular imaging: 3rd biennial clinical trials update

**DOI:** 10.1117/1.JBO.28.5.050901

**Published:** 2023-05-13

**Authors:** Patrick Bou-Samra, Najib Muhammad, Austin Chang, Ritesh Karsalia, Feredun Azari, Gregory Kennedy, Walter Stummer, Janos Tanyi, Linda Martin, Alexander Vahrmeijer, Barbara Smith, Eben Rosenthal, Patrick Wagner, David Rice, Amy Lee, Abdelhafeez Abdelhafeez, Marcus M. Malek, Gary Kohanbash, Wilson Barry Edwards, Eric Henderson, Jane Skjøth-Rasmussen, Ryan Orosco, Summer Gibbs, Richard W. Farnam, Lalitha Shankar, Baran Sumer, Anand T. N. Kumar, Laura Marcu, Lei Li, Victor Greuv, Edward J. Delikatny, John Y. K. Lee, Sunil Singhal

**Affiliations:** a University of Pennsylvania, Perelman School of Medicine, Philadelphia, Pennsylvania, United States; b University of Muenster, Department of Neurosurgery, Muenster, Germany; c University of Virginia, School of Medicine, Charlottesville, Virginia, United States; d Leiden University, Medical Center, Leiden, The Netherlands; e Harvard University, School of Medicine, Boston, Massachusetts, United States; f Stanford University, School of Medicine, Stanford, California, United States; g Allegheny Health Network, Pittsburgh, Pennsylvania, United States; h University of Texas Southwestern Medical Center, Dallas, Texas, United States; i Seattle’s Children’s Hospital, Seattle, Washington, United States; j Saint Jude Children’s Research Hospital, Memphis, Tennessee, United States; k Children’s Hospital of Pittsburgh, Pittsburgh, Pennsylvania, United States; l The University of Pittsburgh Medical Center, Pittsburgh, Pennsylvania, United States; m University of Missouri, Columbia, Missouri, United States; n Dartmouth College, Geisel School of Medicine, Hanover, New Hampshire, United States; o The University of Copenhagen, Copenhagen, Denmark; p The University of New Mexico Medical Center, Albuquerque, New Mexico; q Oregon Health & Science University, Knight Cancer Institute, School of Medicine, Portland, Oregon, United States; r Las Palmas Del Sol Healthcare, El Paso, Texas, United States; s National Institute of Health, Bethesda, Maryland, United States; t University of Texas Southwestern Medical Center, Dallas, Texas, United States; u University of California Davis, School of Medicine, Sacramento, California, United States; v California Institute of Technology, Pasadena, California, United States; w University of Illinois at Urbana-Champaign, Urbana-Champaign, United States

**Keywords:** intraoperative molecular imaging, contrast agents, clinically significant events, precision surgery

## Abstract

**Significance:**

This third biennial intraoperative molecular imaging (IMI) conference shows how optical contrast agents have been applied to develop clinically significant endpoints that improve precision cancer surgery.

**Aim:**

National and international experts on IMI presented ongoing clinical trials in cancer surgery and preclinical work. Previously known dyes (with broader applications), new dyes, novel nonfluorescence-based imaging techniques, pediatric dyes, and normal tissue dyes were discussed.

**Approach:**

Principal investigators presenting at the Perelman School of Medicine Abramson Cancer Center’s third clinical trials update on IMI were selected to discuss their clinical trials and endpoints.

**Results:**

Dyes that are FDA-approved or currently under clinical investigation in phase 1, 2, and 3 trials were discussed. Sections on how to move benchwork research to the bedside were also included. There was also a dedicated section for pediatric dyes and nonfluorescence-based dyes that have been newly developed.

**Conclusions:**

IMI is a valuable adjunct in precision cancer surgery and has broad applications in multiple subspecialties. It has been reliably used to alter the surgical course of patients and in clinical decision making. There remain gaps in the utilization of IMI in certain subspecialties and potential for developing newer and improved dyes and imaging techniques.

## 1 Introduction

According to the World Health Organization, there were almost 18 million patients living with cancer in 2020. It is estimated that over 14 million patients will require oncologic surgery in 2040. This is a 52% increase from 2018 estimations.^1^ Due to the importance of a complete (R0) resection on survival and the increase in detection of early-stage disease, surgical resection remains an important treatment of solid tumors.^2^^,^^3^ Achieving an R0 resection and identifying disease in lymph nodes or synchronous lesions has been shown repeatedly to improve oncologic outcomes and is a mainstay of curative-intent treatment.^4^ However, achieving these margins and finding residual disease during surgery remains a challenge for surgical oncologists. This is mainly due to the inability to see microscopic disease left behind and, at times, uncertainty about involvement of vital structures.

**Fig. 1:**
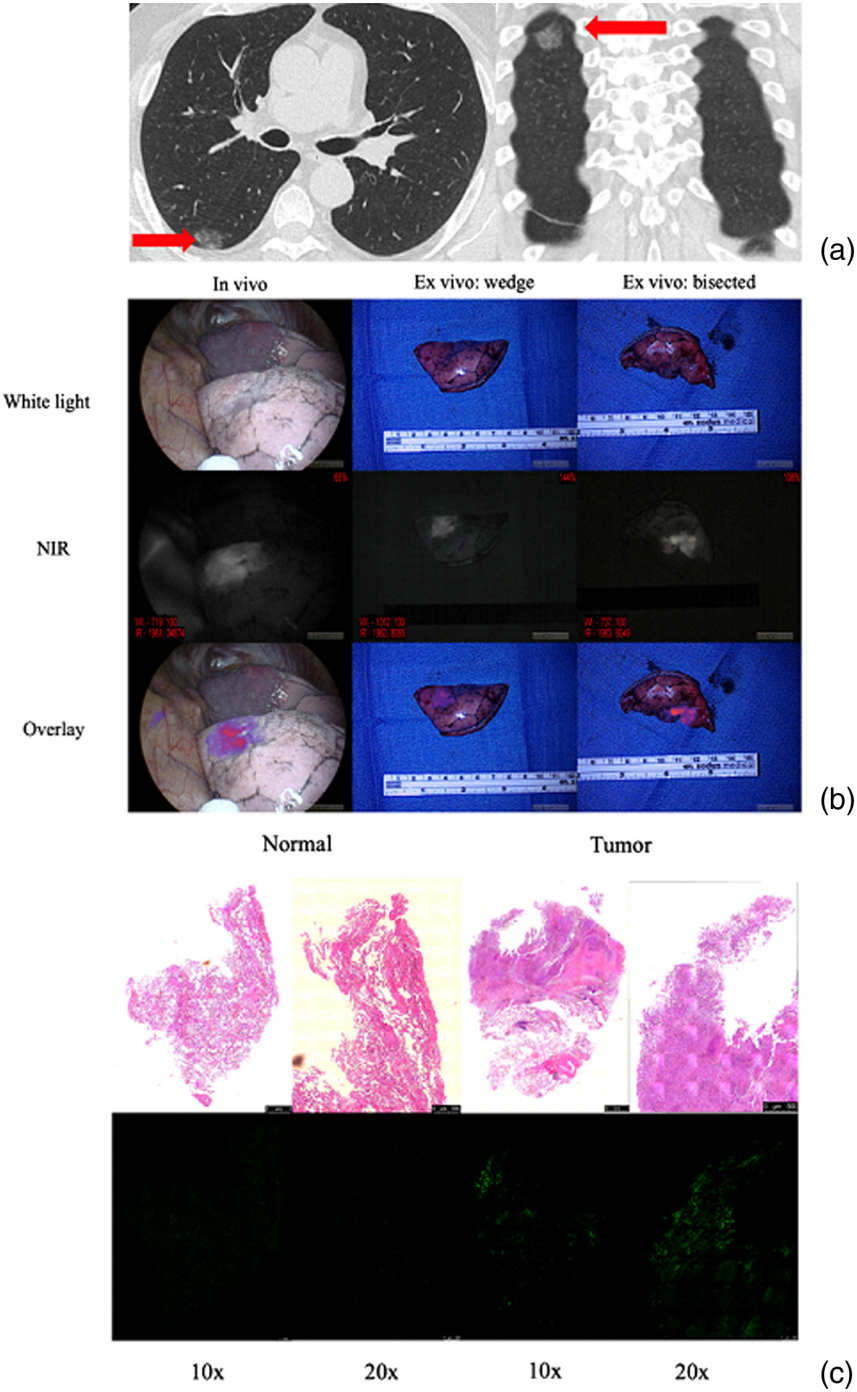
Utilizing IMI for tumor visualization. (a) CT scan shows location of lesion of interest. (b) Lesion assessed initially with standard white light on thoracoscope *in vivo*, then under NIR light to localize lesion. An overlay image is also created by the imaging system. The specimen is then evaluated on the back table and bisected to confirm fluorescence in the suspected area. (c) H&E stain showing tumor and normal with fluorescence microscopy showing the corresponding area of fluorescence. All animal and human research has had the approval of Institutional Review Boards and corresponding committees in each institution.

Intraoperative molecular imaging (IMI) has emerged as a technology to improve intraoperative localization of lesions during cancer surgery (Fig. 1). IMI has been shown to help identifying synchronous disease, detecting occult disease, and confirming a negative margin.^5^ The agents and imaging techniques used in IMI vary. They can be a fluorescent contrast agent that can be nonspecifc, targeted, or activated (Table 1). These are agents that are injected intravenously preoperatively, and when excited with a laser, emit a wavelength that is detected by a photon detector. There are other label-free techniques that leverage the autofluorescent properties of proteins. Other techniques do not rely on a contrast agent but on photoacoustics. However, these methods are still under development. Ideally, surgeons try to preserve as much normal parenchyma as possible, a strategy that is most important in areas where regeneration of the tumor is limited, such as the brain. In these scenarios, IMI delineates the area of interest and specifies resection margins.

**Table 1:** Different tissue dyes stratified into their type: passive targeting, specific targeting, and activatable dyes with mechanisms of actions of each.

Passive targeting	Mechanism	Specific targeting	Mechanism	Activatable dyes	Mechanism
ICG	EPR	Pafolacianine (OTL-38)	Targets cells with overexpression of FRα	Pegsitacianine	pH activated
Methylene blue	Cationic dye attracted to anionic polyphosphates, DNA, RA	SGM-101	Targets CEA receptors	VGT-309	Cathepsin (overexpressed in cancer cells) cleavage of quencher
—	—	Panitumumab-IRDye800	Anti-EGFR antibody	Pegulicianine (LUM015)	Cathepsin and matrix metalloproteinase (MMP)-activated dye
—	—	Affibody (ABY-029)	Anti-EGFR antibody	5-ALA	Prodrug metabolized in tumor cell to produce fluorescent protophyrin IX
—	—	Fluoguide (FG-100)	Targets uPAR	—	—
—	—	Tozuleristide (BLZ-100)	Binds calpactin and MMP-2 that are clustered on glioma cells	—	—
—	—	Dintuximab	Anti-GD2 receptor antibody	—	—
—	—	Bevonescein (ALM-488)	Binds normal human neuron	—	—
—	—	Oxazine derivative (LGW16-03)	Binds normal human neuron	—	—

IMI-guided surgery has three requisites: a targeted fluorophore that can be injected systemically and delivered to a tumor, a laser capable of emitting a desired wavelength, and a camera capable of detecting the fluorescence emitted from the cancer. Over the last two decades, these components have each been developed to identify different targets and have had increased clinical implementation. Although traditional imaging is mostly in the visible wavelengths, IMI relies heavily on the near-infrared (NIR) wavelength because it allows deeper tissue penetration and decreased background fluorescence.^6^ The cameras are also specific as they tend to detect the emitted wavelength from the fluorophore but are typically available in most institutions for more perfusion measurements. Delivering the fluorescent dyes falls into one of three major strategies. The first is passive targeting, which relies on enhanced permeability and retention (EPR), whereby the dye preferentially accumulates in the tumor tissue due to leaky vasculature.^7^ A more specific approach to tumor targeting is to target a specific receptor or enzyme that is overexpressed in tumors. A third class of “activatable” dyes take advantage of tumor-specific microenvironments or enzymes to become activated.

Currently, two agents have already obtained Food and Drug Administration (FDA) approval for use in oncologic resections: 5-aminolevulinic acid (5-ALA) and pafolacianine (OTL-38). 5-ALA has been approved for high-grade gliomas in 2017. Subsequently, pafolacianine was approved for ovarian cancer in November 2021 and for lung cancer in December 2022.^8^^–^^10^ Currently, many other cancer-specific dyes are being investigated under clinical trials. The current research focuses on fine-tuning these dyes to overcome some of the challenges with previous dyes, such as depth penetration and background noise.^11^ Also, there is increased interest in “normal-tissue” dyes that would help not only in oncologic surgery but also in delineating normal anatomy in surgically challenging or reoperative fields. From an imaging standpoint, there is a need to develop better cameras that can decrease background on the one hand and ideally identify several emission wavelengths on the other.

To consolidate the wealth of knowledge and research we have at this time and encourage collaborative growth, a group of 300 researchers and industry partners from all over the United States and the world came together in Philadelphia on November 11, 2022. This was the third biennial IMI conference hosted at the University of Pennsylvania. The goal of the conference was to present the current clinical advances including FDA approvals, phase 3, phase 2, phase 1, and preclinical work pertaining to IMI. The clinicians shared their real-time experience with this technology and demonstrated its direct impact on patient care and oncologic outcomes. There was also a demonstration of the gradual stages of drug development and applications across different specialties. This paper summarizes the main points in the discussions surrounding the most recent advances in IMI.

## 2 FDA-Approved Agents

### 2.1 Summary of Main Points

• A clinically significant event (CSE) is defined as: localization of a tumor that was not detectable by traditional white light or tactile sensation, identification of a synchronous lesion, or identification of a positive margin.
• 5-ALA
  • FDA approved for fluoresce-guided resection of high-grade gliomas and may have applications in ovarian and breast cancer.
  • The triple-LED/loupe system to be a useful adjunct to surgeons who prefer loupes to microscopes during surgical resection.
• Pafolacianine (OTL38)
  • IMI is a valuable adjunct to help achieve maximal cytoreduction and improve patient prognoses in ovarian cancer.
  • Using OTL38 altered the operative plan in 56% of patients with ovarian cancer and led to a more complete debulking in 50.5% of patients.
  • Fifty-four percent of patients with lung cancer undergoing IMI and a wedge or a segmental resection with OTL38 had a CSE; 29% of patients had a change in operative course based on IMI findings.

### 2.2 5-ALA in Europe: Fluorescence-Guided Resection of Meningiomas

In 2017, the US FDA approved 5-aminoleuvulinic acid (5-ALA) for fluorescence-guided resection of high-grade gliomas.^12^ 5-ALA is a prodrug that is metabolized in tumor cells to produce protoporphyrin-IX (PPIX), which is the fluorescent moiety that will remain in the cells. As such, PPIX is not found in the bloodstream, and fluorescence does not leak into normal brain tissue, yielding minimal background signal. With a positive-predictive value (PPV) of 98%, 5-ALA can serve as a guide to the surgeon who can follow the fluorescence in the brain leading to the glioma tissue.^13^ Dr. Walter Stummer, MD, PhD, from Munster University Hospital, provided updates regarding imaging devices, target populations, and expanding indications for the use of 5-ALA (ClinicalTrials.gov, no. NCT00241670).

Dr. Stummer presented new loupe devices (Reveal FGS; Design for Vision, Inc.), which are more closely related to microscopes than exoscopes, allowing the surgeon to view the fluorescence without any intervening cameras.^13^ The loupes include three diodes: white light, 450 nm, and 405 nm (excitation peak of protoporphyrin). In 2021, Dr. Stummer validated the technical measures of the device showing that the performance of the loupes regarding fluorescence discrimination were equivalent to a conventional Kinevo surgical microscope (Carl Zeiss, Meditec). The loupes, however, demonstrated a much stronger fluorescence and background brightness when both systems were compared using an artificial eye for determining fluorescence intensity as seen by surgeons. Furthermore, they provided clinical validation by comparing fluorescence of freshy extracted tissue with both systems and found equivalent signal. While the loupes provided 10× the fluorescence brightness of conventional microscopes, the downside was that the surgeon was unable to visualize deep cavities. As such, the triple-LED/loupe system is a useful adjunct to surgeons who prefer loupes to microscopes during surgical resection.

While the FDA has approved the use of 5-ALA for high-grade glioma resection in adult patients, little is known about its safety and efficacy in pediatric patients.^14^^,^^15^ As such, Dr. Stummer then discussed an ongoing safety trial involving eight institutions across Germany and the United Kingdom. It aims to enroll patients with a wide range of pediatric brain tumors and is currently recruiting additional patients to complete the study.

Finally, Dr. Stummer discussed the expanding indications for 5-ALA guided resection. While meningiomas are commonly benign conditions, maximal resection is critical to prevent recurrence and progression to more malignant grades.^15^ Stummer et al. described the NXDC-MEN-301 phase 3 open-label single-arm study investigating the safety and efficacy of 5-ALA for visualization of meningiomas during resection.^16^ The primary outcome measure for that study is the proportion of participants who have one or more indeterminate tissue of unexpected fluorescence at the end of surgery where 5-ALA fluorescence is consistent with histology. He described the blinding design of the study, i.e., recording all assessments during the study and providing these images to a blinded panel. He explained that the enrollment of subjects is nearly complete. Finally, he mentioned trials for 5-ALA in ovarian cancer and breast cancer, illustrating the potential expanding indications.

### 2.3 Pafolacianine for Ovarian Cancer

Dr. Janos L. Tanyi from the Perelman School of Medicine at the University of Pennsylvania discussed the results of the phase 3 study assessing the efficacy of pafolacianine sodium injection (OTL38) for the IMI of ovarian cancer. OTL38 is a folic acid analog conjugated to an indo-cyanine green-like dye (excitation wavelength of 774 nm and emission wavelength of 794 nm) that works by leveraging the tumor-specific overexpression of folate receptor alpha (FRα). FRα is overexpressed in many solid malignancies, including ovarian cancer, where over 97% of high-grade ovarian cancers are known to overexpress the protein in moderate to high levels.

Dr. Tanyi summarized the phase 1 and 2 studies that identified the optimal dose of OTL38 (0.025  mg/kg) and time window of administration (>1  h before surgery). It also showed that OTL38 helped identify additional lesions that were not detected by visual inspection or white light in 49.3% of patients with ovarian cancer. The phase 3 study was an open-label, single-dose, randomized prospective trial across 11 tertiary care cancer centers. (ClinicalTrials.gov, no. NCT03180307). The primary objective of the study was to confirm the efficacy of OTL38 with intraoperative NIR fluorescence imaging to detect additional FRα+ ovarian cancer lesions that were not detectable by normal white light and palpation. The secondary objectives of the study were to estimate the sensitivity and false-positive rate of OTL38 and assess safety. A total of 150 patients were infused, with 109 patients analyzed for the primary and secondary endpoints, and it was found that 33% of patients had ≥1  FRα+ ovarian cancer lesion detected by intraoperative fluorescence imaging that was not originally planned for resection based on white light and palpation. OTL38 showed an overall sensitivity and false-positive rate of 83% and 32.7%, respectively, with post-hoc analysis showing a sensitivity and false-positive rate of 86.5% and 28.5% when adjusted for high-volume surgeons. There were no drug-related serious adverse events, with the most common events including mild to moderate levels of nausea, vomiting, and abdominal pain. Dr. Tanyi concluded his presentation by sharing investigator reported outcomes in a postprocedural questionnaire, which showed that intraoperative fluorescence led to the revision of the surgical plan in 56% of cases and to a more complete debulking in 50.5% of patients. The phase 3 study results made a compelling argument for the use of OTL38 and intraoperative fluorescence imaging as an adjunct to achieve maximal cytoreduction and improve patient prognoses.

### 2.4 Pafolacianine for Lung Cancer: Enabling Lung Cancer Identification Using Folate Receptor Targeting (ELUCIDATE) Trial

Dr. Linda Martin from the University of Virginia explained the challenge lung cancer surgeons face with the increased adoption of minimally invasive techniques due to less tactile sensation. Furthermore, she identified that localization of subpleural or a deep nodules in the lung and ground-glass opacities is difficult. Another major challenge in thoracic surgery is intraoperative assessment of margins which requires a significant amount of dependence on pathology which is subject to human error.

Dr. Martin summarized the mechanism of action of pafolacianine, OTL38 (On Target Laboratories, West Lafayette, Indiana). She explained that lung adenocarcinomas have a high density of FRα expression.^17^ After undergoing cell line and animal model studies, this drug was first used in a phase 1 study for lung cancer in 2016 and had no serious adverse events.^18^ It was then evaluated in a phase 2 multi-institutional single arm study and showed improved outcomes in 26% of patients.^19^ As such, the decision was made to evaluate the use of pafolacianine for lung cancer in a multi-institutional randomized, phase 3 study (ClinicalTrials.gov, no. NCT04241315).

Patients were eligible if they were suspected to have lung cancer, planned to undergo a minimally invasive surgery, and planned to undergo sub-lobar resection initially with the possibility of proceeding to a lobectomy based on surgeon judgment. Patients (n=112) consented to the study and received a preoperative infusion of pafolcianine 1 to 24 h prior to surgery. Then, they proceeded to the operating room. First, they underwent thoracoscopic evaluation to identify lesions and included using finger palpation if needed and possible. Whether or not a lesion was identified was recorded. Subsequently, the patients underwent a randomization of 1:10 as specified by the FDA, where the NIR camera was not turned on for 1 patient but was turned on for 10. Patients acted as their own controls and all results were confirmed by the pathology lab. There were 12 participating institutions: The University of Pennsylvania, University of Pittsburgh Medical Center, Cleveland Clinic, Mayo Clinic, University of Iowa, Allegheny General Hospital, MD Anderson Cancer Center, Beth Israel Deaconess Medical Center, University of Virginia, University of Michigan Medical Center, Stamford Medical Center, and Swedish Cancer Institute.

The primary endpoint was identification of a CSE defined as: either localization of primary nodule that was not detected under normal light or palpation using standard techniques or identification of a synchronous lesion that was not detected under normal light or palpation using standard techniques, or identification of a positive or close margin that was within 10 mm of the surgical staple line when the specimen is evaluated on the back table. The latter helped surgeons to decide whether the margin was close and there was a need to proceed with a wider resection. In terms of demographics, the patients were the typical cohort seen with smoking history (around 70% current or former), mostly adenocarcinoma (typical of peripheral tumors) and 19% with metastatic disease from non-lung primary, such as colorectal cancer or sarcoma. The surgical procedures were wedge, segmentectomy, lobectomy, or no resection (multiple nodules not amenable to surgical resection). Half were robotic and half were traditional video-assisted thoracoscopic surgery. The drug was mostly safe and tolerable. The most common side effects were nausea or vomiting, hypertension, and hypotension. Eleven had infusion-related reaction and four did not finish the infusion because they had a rash. There were no long-term threatening side effects from the drug infusion.

The results of the study showed that 54% of CSE were identified and there were more than one CSE in some patients. 29% of patients had a change in operative course based on IMI findings. For localization, 19% of the primary lesions were not identified with white light but were identifiable with NIR. In 9% of patients, a second or third lesion was identified, the majority of which were outside the planned area of resection. In 38% of patients, the margins assessed by NIR were within close proximity of the planned margin. This was 84% concordant with pathology readings. The secondary endpoint included the positive predictive value of pafolacianine: 99% in identifying primary lesions and 40% for synchronous lesions. The false positives observed were granulomas, intraparenchymal (IP) lymph nodes, and anthracotic nodules.

## 3 Phase 3 Trials

### 3.1 Summary of Main Points

• CEA-targeted fluorescent probe (SGM101)
  • Safe and feasible for detection of colorectal cancer in patients with an R1 resection and allows for a guided resection.
  • Valuable adjunct in recurrent colorectal cancer with increased scarring and difficult to visualize operative fields.
• Pegulicianine
  • In breast cancer, it improved tumor prediction in shave margin compared with routine pathological assessment of the lumpectomy specimen.
  • Pegulicianine-guided surgery reduces the need for re-excision by 19% (Table 2).

**Table 2:** Fluorophores under phase 3 clinical trials.

Presenter	Tracer	Target tumor	Mechanism of action
Alexander L. Vahrmeijer, MD	SGM-101	Colon cancer	Fluorescent anti-CEA antibody
Barbara L. Smith, MD, PhD	Pegulicianine (LUM015)	Breast cancer	Cathepsin and MMP-activated fluorescent dye that accumulates in the tumor bed because of a polyethylene glycol side group

### 3.2 CEA Use for Colon Cancer in a European Phase 3 Trial

Dr. Alexander Vahrmeijier from Leiden University Medical Center (LUMC) presented that IMI was first implemented in 2009 for SLN in breast cancer at LUMC, but then IMI transitioned to colon cancer in 2019. In their study, the group utilized the Artemis and Spectrum fluorescence imaging systems (Quest Medical Imaging, Middenmeer, The Netherlands). An anti-carcino-embryonic antigen (CEA) was chosen to be tagged to a fluorophore because it is expressed on around 80% to 90% of all colorectal cancer. Its expression is also not affected by radiation therapy (RT) or chemotherapy. Initially in the phases 1 and 2 trial, 10 mg SGM-101 (dye-targeted against CEA) was injected 4 days prior to the surgical procedure and was able to detect the primary colonic tumor along with the metastatic deposit in the liver.^20^ Intraoperatively, tumors that were visible under white light are then observed under NIR and a fusion image of both were then seen intraoperatively as well. This allowed to visualize the gross resected margins in real time. They then confirmed on pathologic evaluation.

In recurrent rectal cancer cases, the operative field has increased scarring and it is more difficult to distinguish areas of concerns. The use of NIR allows to visualize areas that had an R1 resection and allows a guided resection. It also allows the visualization of distant metastatic colorectal cancer deposits in the lung. In the phase 2 trial, there were 37 patients enrolled with primary and recurrent colorectal cancer. As a result of NIR, eight additional malignant lesions were identified in six patients, and there was a change of surgical plan in 24% (9/37) patients where seven needed additional resections and two required downstaging in treatment.^21^ SGM-101 was able to impact a change in surgical plan in one of four patients.

Given the promising results in the phases 1 and 2 trial, the group is currently performing a phase 3 trial. The study aims to enroll a total of 300 patients between Italy, Germany, the Netherlands, and the United States. Indications include clinical T3 or T4 colorectal tumors and recurrent disease with peritoneal carcinomatosis. It is randomized in a 1:4 fashion where one patient does not receive SGM-101 and four others do. The process is completely blinded to the surgeon and unblinding only happens prior to the case. At that time, the surgeon would either proceed with NIR inspection, resect, then use NIR to rescan the area and resect further if need be or undergo the standard surgical procedure without NIR (ClinicalTrials.gov, no. NCT04737213).

On a different note, Dr. Vahrmeijer explained new optical advances in developing a dual channel camera that can detect multiple wavelengths. For instance, the Artemis and Spectrum fluorescence imaging systems (Quest Medical Imaging, Middenmeer, The Netherlands) at this time can detect the primary tumor with SGM-101 in the colon. When the channel is changed to 800 nm, it can image the ureter as well.

### 3.3 Protease Activated Dye in Breast Cancer Surgery: Phase 3 US Trial

Dr. Barbara L. Smith from Massachusetts General Hospital shared the results of two multicenter randomized trials assessing the utility of pegulicianine guided surgery for breast cancer. Assessing breast lumpectomy margins is uniquely challenging due to complex ductal anatomy and is time consuming, often requiring multiple days to receive the results of the lumpectomy and perform additional surgery when margins remain positive (20% re-excision rate). Furthermore, standard pathology often has limited accuracy correlating positive lumpectomy specimen margins with residual tumor in the surgical cavity. Thus, the ideal breast lumpectomy margin assessment tool is one that has a high sensitivity for tumor detection, provides a rapid assessment, and identifies residual tumor inside the cavity wall.

Pegulicianine (LUM015) is a cathepsin and MMP-activated fluorescent imaging agent that preferentially accumulates in the tumor bed. Pegulicianine is administered at a dose of 1.0  mg/kg in a 3-min IV push ∼2 to 6 h prior to surgery. After removal of the main lumpectomy specimen, the surgical cavity is assessed using a direct visualization system (Lumicell, Inc.), which includes a handheld intracavity probe that can detect the fluorescence emission of pegulicianine at a wavelength of 670 nm. The imaging system has a field of view of 2.6 cm diameter area in 1 s and currently adds <10  min to the surgical procedure time.

Dr. Smith’s team conducted an initial feasibility study with 45 breast cancer patients and 569 cavity margin images, which revealed promising results with a sensitivity of 84% for tumor in cavity wall, specificity of 73%, and tumor : normal signal ratios between 3.52 and 5.69. This initial study showed that the fluorescence signal was not affected by tumor histology (DCIS versus IDC versus ILC), breast density, or menopausal status. The study also showed that pegulicianine had no impact on tumor histopathology or receptor determination. The next major study conducted was a multicenter feasibility study that included 234 patients over 16 US sites, with three to five cases per surgeon. Patients were females with either primary invasive breast cancer or DCIS undergoing lumpectomy. The multicenter feasibility study showed a sensitivity of 69% for predicting tumor in shave margin, compared with a sensitivity of 38% for routine pathological assessment of the lumpectomy specimen. The study also showed a specificity of 70%, compared with 91% for routine pathological assessment of the lumpectomy specimen, for predicting tumor in the shave margin. Pegulicianine-guided surgery reduces the need for re-excision by 19%, which would have been closer to 32% if all the surgeons in the study had excised all lumpectomy cavity areas with fluorescent signal^22^ (ClinicalTrials.gov, no. NCT04440982).

The next multicenter trial that Dr. Smith’s team conducted was a prospective pivotal study, which was a randomized 10:1 pegulicianine-guided surgery : control trial comprised of 406 patients across 14 US sites. The patients were again females with primary invasive breast cancer or DCIS undergoing lumpectomy. Though the results of the study have not been published yet, Dr. Smith highlighted that pegulicianine reduces the need for second surgeries for positive margins and helped remove tumor missed by standard lumpectomy. On average, one additional shave was taken when surgery was guided by pegulicianine, with an average of 10 cc of additional tissue taken. Overall, Dr. Smith highlighted that pegulicianine had a 98% negative predictive value, emphasizing its utility in breast lumpectomies. The next multicenter study will assess the extent of residual tumor remaining after pegulicianine-guided surgery compared with standard lumpectomy. It will also examine the influence of neoadjuvant endocrine or chemotherapy on pegulicianine-guided surgery. This study is currently undergoing patient accrual.

## 4 Phase 2 Trials

### 4.1 Summary of Main Points

• Panitumumab
  • Drains from head and neck tumors to sentinel and tumor-positive lymph nodes;
  • Combining antibody-based tracers with traditional F18-FDG PET/CT may offer a safe, low-cost addition for improved preoperative localization of true positive lesions and lymph nodes.
• VGT-309
  • Is a quenched-activity-based probe that is activated by cathepsins in tumor molecules.
  • VGT-309 has had promising preclinical and phase 1 data for its use in the differential detection of lung cancer.
• Pegsitacianine
  • Is a pH-activatable dye that gets activated in the acidic tumor microenvironment.
  • Pegsitacianine detects residual disease that otherwise would have been left behind in standard of care surgery and helps in the completeness of cytoreduction surgery in peritoneal carcinomatosis.
  • Pegsitacianine can detect ground-glass opacities and alter the operative course in lung cancer patients.
  • pH activatable dyes have the advantage of being tumor agnostic and hence have a broader application (Table 3).

**Table 3:** Fluorophores under phase 2 clinical trials.

Presenter	Tracer	Tumor type	Mechanism of action
Eben Rosenthal, MD	Panitumumab	Head and neck	Fluorescent anti-EGFR antibody
Sunil Singhal, MD	VGT-309	Lung cancer	Cathepsin-activated fluorophore
Patrick Wagner, MD	Pegsitacianine	Peritoneal carcinomatosis	pH-activatable probe
David Rice, MB, BCh	Pegsitacianine	Lung cancer	pH-activatable probe

### 4.2 Anti-EGFR Antibody Imaging Strategies for Real-Time Surgical and Pathologic Guidance

Dr. Eben Rosenthal’s group is currently evaluating the use of panitumumab conjugated to IRDye800 for NIR surgical imaging in two settings: intraoperative real-time assessment with an open-field system (Novadaq, Burnaby, Canada) and back table specimen assessment with a closed-field system (LI-COR Biosciences, Lincoln, Nebraska). His group has shown that the antibody-based tracer is specific for tumor cells and EGFRs at the whole tumor and pathologic level.^23^ Data were presented to support IMI for detection of satellite lesions, determining deep margin status, and identifying residual subclinical disease.^24^ This technology identified areas suspicious for residual tumor, reducing the sampling error limits associated with frozen section analysis when sending tissue to pathology for confirmation.^23^^,^^25^

Systemic administration of panitumumab-IRDye800 is also useful for staging of solid tumors and identification of metastatic lymph nodes. Dr. Rosenthal’s clinical studies have suggested that the anti-EGFR-labeled tracers may drain from the tumor to sentinel lymph nodes (SLN) by indirect or passive drainage from the primary tumor (just as if it were directly injected). This is in addition to the direct (hematogenous) targeting tumor-containing lymph nodes. His group has validated that fluorescence intensity is dose dependent and metastatic nodes can be differentiated from benign nodes. They are able to accurately stage the tumor by examining the three lymph nodes with the highest fluorescence intensity, indicating that molecular guided surgery has the potential to improve the workflow of staging and SLN biopsies.^26^^,^^27^ Data were also provided to support the use of anti-EGFR antibodies for tumor detection using PET-based imaging. In a trial using panitumumab conjugated to Zirconium89, it was found that Zr89-panitumumab PET/CT imaging could be used to rule out false positives identified on F18-FDG PET/CT^28^ (ClinicalTrials.gov, no. NCT02415881).

### 4.3 Intraoperative Molecular Imaging of Lung Cancer with Cathepsin Imaging

Dr. Sunil Singhal from the University of Pennsylvania describes the three mechanisms of action of IMI tracers: nonspecific or passive such as ICG, targeted receptors such as folate receptor, and the third category is activatable probes as described by Dr. Smith earlier. The latter are only activatable in the tumor microenvironment and are nonfluorescent if left alone. Those can be enzyme-activated where the molecule is formed by a fluorochrome, a linker, and a quencher. The quencher prevents the probe from fluorescing until cathepsin (one of the enzymes selectively upregulated in tumor cells) comes in contact and cleaves the quencher, thereby allowing the fluorochrome to become visible. The other mechanism involved is molecular binding at which point the tracer binds the receptor, endocytosed and transported to the lysosome where it is activated. These enzymes include cathepsins B/L, MMP, and beta galactosidases. The advantages of activatable probes are the ability to obtain excellent resolution, undergoing a quick enzymatic chemical reaction, having less side-effects due to low doses used, having favorable pharmacokinetics, and covalently binding to the cathepsin so it is retained at the site.

The cathepsins are a family of 11 proteases normally found in the lysosomal degradation system and are overexpressed in cancer cells, tumor-associated macrophages, and tumor microenvironments. Cathepsin is found in multiple cancer cell types including brain, lung, or prostate. Preclinical studies showed that cathepsins are expressed in multiple lung cancer cell lines: lung adenocarcinoma, large cell, adenosquamous carcinoma, and squamous cell. This is advantageous as lung cancer has the most varied cancer subtypes.^29^

The VGT-309 (excitation 789 nm, emission 814 nm) (Vergent Bioscience, Minneapolis, Minnesota) molecule was developed by Dr. Matt Bogyo at Stanford.^30^ A phenoxymethyl ketone electrophile covalently binds cathepsin and makes it irreversible. There is also a quencher that prevents ICG from fluorescing unless the quencher is cleaved. Since the fluorophore is ICG, conventional ICG scopes can be used and those tend to be readily available at most centers. In preclinical studies, the fluoresce of VGT-309 was found to be dose dependent in mice and inhibited when a cathepsin inhibitor is added; 4  mg/kg at 24 h was found to be ideal dosing and timing. The dye is renally excreted and has minimal background except for the spleen and kidney. Cathepsin was also found both in tumor and tumor-associated macrophages.^29^

Then, in collaboration with Dr. David Holt at the Penn School of Veterinary Medicine, canine IMI studies of lung cancer were performed. Intraoperative imaging with white light was compared with NIR and these were confirmed on pathologic specimens. The tumor did take up the dye and lit up.^29^ This was followed by a phase 1 study that was done in Australia looking at safety and tolerability. The higher doses had a long half-life as expected while being well tolerated with minimal side effects. A follow-up phase 2 study was done with 27 patients and the goal was to look at timing (data unpublished at this time). Dr. Gavin Wright in Australia found that the 0.05-mg/kg dose was too low, and they needed to administer 0.16 and 0.32  mg/kg to visualize the tumor better.

At this time, there is an ongoing phase 2 efficacy study at The University of Pennsylvania. The dose currently being used is the 0.32  mg/kg because the tumor is better visualized at this dose, had enough of a half-life to be administered the day before surgery, and a minimal toxicity profile. It is a single arm study that will include 40 patients and the endpoints are identifying CSEs.

### 4.4 pH-Sensitive Dyes in Peritoneal Surface Malignancy

Dr. Patrick Wagner from the Allegheny Health Network presented data on pegsitacianine (OncoNano Medicine Inc., Southlake, Texas), a pH-sensitive molecular imaging agent in a phase 2 study on peritoneal carcinomatosis. Peritoneal metastasis is a pattern of metastatic cancer from various primary sources, including appendiceal, ovarian, uterine, gastric, and colon, with a high recurrent rate following surgery. Preoperative scans can underestimate the disease burden and surgeons are limited to palpation and visual cues to detect all metastatic lesions in the abdomen. Initial disease volume and completeness of surgical cytoreduction correlate with improved patient outcomes, where a cytoreductive score is used to account for no disease present. Given these limitations, there has been an interest in the development of intraoperative imaging. It allows surgeons to get instantaneous feedback, is easy to use, and allows opportunities to detect residual disease.

Pegsitacianine is activated in the acidic tumor microenvironment. It is a micellar fluorescent molecule composed of several hundred amphiphilic unimers, which are composed of a hydrophilic and hydrophobic segment to which ICG is conjugated. Self-assembled into micelles in aqueous solution at physiologic pH, the ICG is sequestered and quenched in the core of the micelle but disassembles in the acidic tumor microenvironment found in nearly all solid cancers, turning the fluorescence ON. The phase separation and cooperativity of the unimers leads to an all-or-nothing binary fluorescence response.^31^^,^^32^ It was evaluated in a phase 1 study in the Netherlands, where it was found to be well tolerated. It was able to detect esophageal, head and neck, breast, and colon cancer. It identified nine positive margins and two instances of occult disease. A phase 2 study was then designed. It addressed early imaging feasibility at times <24  h postdose administration, expansion of range of tumor types to include prostate and ovarian cancer, and performance in head and neck squamous cell carcinoma at 1  mg/kg 24 h postdose administration.^33^

There is an ongoing phase 2 study in peritoneal metastases. The primary objective of this study is to determine if the administration of pegsitacianine (1  mg/kg) prior to surgery results in a CSE as previously described or alters the completeness of cytoreduction score. The secondary objectives include the demonstration of an acceptable safety profile, reliable sensitivity, specificity, negative predictive values, and positive predictive values at the level of the individual specimens. The study design is as follows: at day 0, pegsitacianine is infused 24 to 72 h prior to surgery at a dose of 1  mg/kg and rate of 10  cc/min. Then, at the day of surgery, the patients undergo a standard of care surgery. This is followed by the imaging of 10 suspected tumor nodules and five areas of normal tissue, examination of peritoneal cavity with NIR camera and excision of additional disease. The pathologist then performs a histologic evaluation of the specimen and calculations of the cytoreduction completion score and findings of CSE (ClinicalTrials.gov, no. NCT04950166).

To date, 32 patients have undergone imaging during surgery with pegsitacianine. Disease was detected in six tumor types: appendiceal, colorectal, mesothelioma, endometrial, pancreatic, and ovarian. There was no significant difference in detecting mucinous versus nonmucinous disease. The drug was well tolerated and there were no drug-related adverse events; 27% of patients had an infusion-related transient reaction restricted to flushing. The tumor was able to take up the dye and activate it. Small tumor deposits were detected by pegsitacianine with healthy tissues showing little to no activation. The anticipated background fluorescence was observed in the liver, spleen, small bowel, and lymph nodes. It was also able to consistently detect residual disease and alter complete cytoreduction score. There were 466 samples with a sensitivity of 86%, NPV 78%, and FPR 27%. In terms of imaging metrics, the mean fluorescent activity of the tumor was 2.49 compared with 1.6 for normal tissue, which was found to be statistically significant. All the tissues were imaged in a background of normal mesenteric tissues.^34^ There are several camera systems on the market compatible with ICG and pegsitacianine. In this study, the Visionsense Iridium camera system (Visionsense EleVision) was used. Other open-air ICG compatible cameras have been used with pegsitacianine in the current and completed clinical trial.

In conclusion, pegsitacianine aids in the visualization of residual disease that otherwise would have been left behind in standard of care surgery. It also alters the completeness of cytoreduction surgery which is correlated with improved outcomes. Some limitations include the ability for surgeons to differentiate lymph nodes from residual disease and that there is a learning curve to learn how to interpret fluorescence. In terms of potential future applications, Dr. Wagner explained how it can be used to detect primary malignancies of peritoneal origin and in using this technology for laparoscopic staging of disease and determining PCI score.

### 4.5 pH-Sensitive Dyes in a Phase 2 Lung Cancer Trial

Dr. David Rice from the University of Texas MD Anderson Cancer Center presented work on the use of pegsitacianine for IMI in lung cancer. He explained that the need for a tumor agnostic, activatable dyes stems from the fact that not all lung cancers express targetable receptors. For example, if an agent targets folate receptors, it will pick up adenocarcinomas more so than squamous cell carcinomas, which express the folate receptor less.^35^ As such, a dye that is tumor agnostic was needed and smart tumor pH-responsive optical probes were developed.

Most of the preclinical lung cancer work was done in Dr. Singhal’s lab at the University of Pennsylvania. Work was initially done to characterize the ideal pH and the time needed to activate the dye. The ideal pH was found to be <6.5. In murine studies, athymic mice were injected with human cell line KB into their flanks. Pegsitacianine was then administered via tail vein injection at various doses. It was found that 24 h was enough time for most optimal uptake. The MFI and tumor-to-background ratio (TBR) was not different between 2 and 4 mg but was superior to the 1-mg dose. The biodistribution to other organs showed that mostly the liver and the spleen took up the drug but less than the tumor did. Importantly, the lungs themselves had very low background uptake. Next, to see if this may be used across tumor cell types, AC and SCC of lung origin into athymic mice. Both showed good uptake of the drug and fluoresced. The tumors were excised and analyzed for uptake within the tumor. The drug appeared to be concentrated in the periphery of the tumor and not the center. The reasons mostly likely are related to the initial uptake of the agent with relatively poor vascularity in the center of the tumor.

The phase 1 study was done in the Netherlands and involved 30 patients. It showed uptake across a broad range of histologic subtypes: HNSCC, breast cancer, colorectal cancer, and esophageal cancer. At this time, there is an ongoing phase 2, single-dose, open-label study to evaluate diagnostic performance and safety of pegsitacianine in patients undergoing routine surgery for suspected lung cancer. This single-arm study is designed to include 40 patients infused 1-mg/kg pegsitacianine 24 h prior to surgery. It is a multi-institutional study involving MD Anderson Cancer Center and The University of Pennsylvania Perelman School of Medicine. Inclusion criteria are any patient with a lung mass suspicious for lung cancer and age 18 to 85. Primary endpoints are similar to previous studies and aim to determine if there were CSEs. The secondary endpoints are safety, toxicity, sensitivity, specificity, PPV, and NPV. For example, a GGO in the upper lobe of the right lung is hard to palpate and visualize. Intraoperatively, when viewing this lesion, there is no tethering of the surrounding normal tissues indicative of a deeper tumor. Using NIR, pegsitacianine is able to localize the otherwise occult disease. Also, pegsitacianine was able to guide decision-making between a bilobectomy versus total pneumonectomy by providing additional fluorescent information on the tumor’s extent and missed lesions under white light.

PH activatable dyes have the advantage of being tumor agnostic and hence have a broader application. Meanwhile, pegsitacianine is visible using commonly available camera systems tuned to detect ICG. The preclinical data suggest efficacy with tracer uptake. At this time, patients are being recruited for the phase 2 study (ClinicalTrials.gov, no. NCT05048082).

## 5 Pilot and Phase 1 Clinical Trials

### 5.1 Summary of Main Points

• Affibody
  • ABY-029 follows EGFR very closely in terms of staining sarcomas.
  • ABY-029 does not bind necrotic cores that are common in sarcomas but combination with ICG allows for more homogeneous fluorescence signal.
• Urokinase
  • Urokinase-type plasminogen activator receptor (uPAR) is an extracellular receptor expressed on cancer cells that is targeted by FG-100.
  • There are ongoing phase 2 trials that have been initiated at two Scandinavian centers to evaluate the use of FG-100 in gliomas.
• Second window ICG (SWIG)
  • SWIG can predict accurately what postoperative MRI can in terms of adequate gross total resection.
  • SWIG allows the visualization of tumor tissue in the brain 24-h after the initial infusion of ICG.
  • It is used for brain metastases, intracranial meningiomas, and skull base tumors (Table 4).

**Table 4:** Fluorophores under phase 1 clinical trials.

Presenter	Tracer	Tumor type	Mechanism of action
Eric Henderson, MD	Affibody (ABY-029)	Sarcomas	EGFR binding (affibody)
Jane SkjothRasmussen, MD, PhD	Fluoguide (FG-100)	Gliomas	uPAR-targeted dye
John Y.K. Lee, MD, MSCE	ICG	Meningiomas, gliomas, brain metastasis, skull base tumor	EPR effect

### 5.2 Antiepidermal Growth Factor Receptor Affibody: Design and Implementation of a Novel Targeted Tumor Dye

Sarcomas are <1% of all cancers and derived from mesoderm, the middle germ layer. Unlike other IMI applications where intralesional tumor removal is the standard of care (e.g., glioma), sarcomas should be excised with a wide margin and the tumor is never directly visualized during surgery, a technique called wide local excision. However, some of the largest series report up to 22% positive margin rates.^36^ This requirement creates new challenges for applying IMI to sarcomas.

In an effort to leverage IMI using a small-molecule NIR probe, an academic-industrial collaboration (Affibody, LI-COR, UAB, Dartmouth) created an affibody-based probe intended for day-of-surgery dosing (ABY-029). ABY-029 was initially designed for gliomas but applied to H&N cancers and sarcomas. ABY-029 consists of an anti-EGFR affibody tagged to IRDye-800cw. It was adapted to sarcomas because around 60% of sarcomas overexpress EGFR, and higher-grade tumors tend to present higher concentrations of EGFR.^37^

ABY-029 synthesis began in 2013. Preclinical toxicity testing occurred between 2015 and 2016, demonstrating no toxicity at levels up to 1000× the human-equivalent microdose. ABY-029’s no observable adverse effect limit (NOAEL) was determined to be 24.5  mg/kg in mice, the human equivalent is 237 mg or 3.95  mg/kg for a 60 kg person; there was strong binding affinity and no observed toxicity.^30^ In preclinical work, fluorescence was correlated strongly to EGFR expression, with contrast peaks 4 to 8 h after administration. TBR contrast for ABY-029 was generally 3 to 4 compared with adjacent muscle or adipose tissue. When evaluated by immunohistochemistry (IHC), ABY-029 correlated strongly to EGFR. However, ABY-029 binding in necrotic regions was low. In an effort to improve whole-tumor contrast, ICG was used in a murine xenograft model and demonstrated improved contrast in necrotic tumor regions.

One concern about use of ABY-029 in humans is the potential effects on EGFR expression by standard neoadjuvant sarcoma therapies, usually doxorubicin-based chemotherapy and/or external beam RT. Hence, EGFR+ and EGFR– sarcoma human xenografts were exposed to doxorubicin chemotherapy, radiation, or both. Neither exposure appeared to have a negative effect on EGFR expression or ABY-029 TBR, with RT associated with increased EGFR expression and increased TBR.

A phase 0/1 dose escalation trial was undertaken in humans. Patients underwent day-of-surgery ABY-029 dosing and then tumor resection. Following excision, tumors were brought to pathology, inked, and underwent bread loaf sectioning. One or two sections then underwent low-resolution scanning to identify three areas of high signal and three areas of low fluorescence. These areas of high and low fluorescence were then biopsied, and the resulting tissue specimens underwent high-resolution scanning followed by slide preparation and histopathological evaluation. ABY-029 fluorescence showed strong, linear correlation with the percent area of EGFR (p<0.0001). There were no adverse or toxic events related to ABY-029 administration.

In summary, IMI for sarcomas appears to be feasible and should be pursued in more robust study. Based on the heterogeneity of sarcomas and their propensity for central necrosis due to large size and rapid growth, the use of multiple fluorophores within a similar wavelength will likely provide better whole-tumor labeling. Furthermore, the use of both long- and short-wavelength probes would likely provide advantages for the localization of the tumor (long wavelength) versus margin assessment (shorter wavelength).

### 5.3 uPAR-Targeting Dye (Fluosuide) for Surgery of Gliomas

Dr. Jane Skjøth-Rasmussen from Rigshospitalet in Copenhagen, Denmark, discussed the utility of FG001 for the intraoperative visualization of malignant gliomas. FG001 is a novel uPAR targeting dye. uPAR is an extracellular receptor expressed on cancer cells and/or activated stromal cells, allowing uPAR to serve as a biomarker of cancer. Normally, there are low levels of uPAR expression in healthy tissue and high levels of uPAR correlate with an aggressive cancer phenotype. FG001 is a fluorophore comprised of a uPAR-binding peptide covalently bound to indocyanine green (ICG), allowing FG001 to bind specifically to uPAR while maintaining the same fluorescence properties as ICG (excitation around 789 nm and emission around 814 nm).

Dr. Skjøth-Rasmussen discussed the first in-human study using FG001 in patients with malignant gliomas. A total of 40 patients (36 of whom had high grade gliomas) were included in this open-label nonrandomized multicenter phase 1/2 clinical trial. The primary aim of the study was to evaluate the safety and tolerability of single intravenous doses of FG001 and establish the optimal dose for imaging. The secondary objectives were to determine the pharmacokinetic profile of FG001 and evaluate the efficacy of FG001 in patients undergoing tumor resection. The results of the study revealed that the administration of FG001 16+ h prior to surgery allowed for better tumor discrimination compared with the administration of FG001 closer to surgery, with a dosage of 36 mg having the most favorable TBR. Upon histopathological confirmation, FG001 had a specificity of 100%, sensitivity of 79%, PPV of 100%, and NPV of 58%. There were a total of four FG001-related adverse events in the study, three of which were grade 1 and one of which was grade 2.^38^

At this time, there is an ongoing phase 2b trial that has been initiated at two Scandinavian centers. This trial is comparing the use of FG001 against 5-ALA for influencing surgical strategy in patients with high-grade gliomas. Future trials will also look to assess the utility of FG001 in low-grade gliomas.

### 5.4 TumorGlow™ Second Window ICG in Brain Tumors

In 2016, Lee et al. reported data on the visualization of tumor tissue in the brain 24-h after the infusion of high dose ICG (TumorGlow™).^39^ The mechanism is the EPR effect whereby the disrupted tumor blood–brain barrier (BBB) allows small molecules such as ICG to accumulate in tumor tissue and be retained due to lack of drainage. This is similar to the retention of gadolinium contrast in tumor tissue. Dr. John Y.K. Lee, Professor of Neurosurgery and Clinical Director for the Center for Precision Surgery at the University of Pennsylvania, explained the technique’s unique benefits and applications. First, this SWIG guides the resection as early in the surgery as the corticectomy. Because the NIR signal can be visualized in some cases through the brain and dura, this allows the surgeon to choose the shortest path to the tumor. Other dyes such as 5-ALA and previously discussed peptide-based dyes only illuminate the periphery of the tumor. This may be due to the necrotic core of the tumor, where the tumor grew too fast for the blood supply. With SWIG, the central necrotic core retains the ICG, creating a robust signal of the entire tumor. This ICG signal can also be seen in areas of radiation necrosis as well.

To date, 374 cases have been done using TumorGlow™ for CNS tumors of many different histologies. High-grade gliomas have had high signal-to-background ratio (SBR) through both cortex (SBR=3.54) and dura (SBR=4.18) with high sensitivity (94.35%) and positive predictive value (95.12%). Furthermore, SWIG has 91% overall accuracy in predicting postoperative MRI by imaging the tumor cavity at the end of resection. ^40^ This technique can be used for brain metastases, intracranial meningiomas, and skull base tumors, among others. One of the limitations is visualization of melanoma metastases (ClinicalTrials.gov, no. NCT03262636).

## 6 IMI in Pediatric Patients

### 6.1 Summary of Main Points

• Tozuleristide (tumor paint)
  • “Tumor paint” selectively binds molecules on glioma cells and is useful in primary and recurrent resections.
  • Specialized imaging systems are needed for ICG visualization in the pediatric patients.
• ICG
  • In ICG-guided surgery for pediatric renal tumors, there was a pattern in which tumors were hypofluorescent and adjacent healthy tissue was avidly fluorescent.
  • ICG is being investigated for positive lymph node detection and retroperitoneal-guided resections of pediatric malignancies.
• Dintuximab
  • The use of dinutuximab (an FDA-approved anti-GD2 antibody) radiolabeled with indium-111 and conjugated to IRDye800CW for NIR surgical navigation is under preclinical evaluation.
  • Anti-GD2 tracer had increased uptake in tumors with a signal detection that was inversely correlated with tumor depth (Table 5).

**Table 5:** Fluorophores in pediatric patients.

Presenter	Tracer	Tumor type	Mechanism of action
Amy Lee, MD	Tozuleristide (BLZ-100)	Gliomas	Binds calpactin and MMP-2 that are clustered on glioma cells
Abdelhafeez Abdelhafeez, MD	ICG	Multiple tumor subtypes and lymph nodes	EPR effect
Marcus Malek, MD	Dintuximab (Anti-GD2 probe)	High-risk neuroblastoma and osteosarcoma	GD2-specififc tracer

### 6.2 Tumor Paint™ Imaging: Pediatric Brain Tumors Phase Two

In pediatrics, brain tumors are the leading cause of cancer death and extent of resection remains the single best predictor of survival and cure in this population.^34^ Dr. Amy Lee, the Chief of Pediatric Neurosurgery at Seattle Children’s Hospital, discussed the investigational agent Tumor Paint™ for maximally safe brain tumor resection. Tozuleristide, also known as BLZ-100 or “Tumor Paint,” is a biconjugate of a derivative of chlorotoxin, a small protein that is thought to selectively bind to targets such as calpactin and MMP-2 that are clustered in lipid rafts on the surface of glioma cells and CG.^41^^,^^42^ These two components along with mannitol that is included in the formulation allow the drug to cross the BBB to selectively bind to tumor cells. This leads to long-lasting intracellular accumulation that can be detected in the NIR range. Tozuleristide can be dosed the same day or the day before surgery with no change in mean fluorescence intensity, offering the surgeon flexibility in terms of workflow.

Since its development in 2011, tozuleristide has been shown to illuminate multiple types of cancer (e.g., basal cell carcinoma and melanoma in the skin, high-grade and low-grade tumors of various types in the brain, and early-stage and invasive cancers in the breast) in phase 1 and phase 2 trials that have enrolled over 220 patients.^43^^–^^45^ For central nervous system tumors, these trials have demonstrated that tozuleristide is useful in adult and pediatric tumors with specificity for both high-grade and low-grade gliomas, can be useful in primary and reresection, has a short serum half-life which minimized background signal, demonstrates intracellular accumulation that lasted for longer surgeries (i.e., >10  h), and can be visible beyond overlying tissue due to NIR fluorescence that has better depth penetration. Because tozuleristide uses doses of ICG in the nanomolar range, as opposed to millimolar range used in vascular angiography, specialized detection devices such as the Canvas imaging system with increased sensitivity are required.

At the end of 2018, Dr. Lee and colleagues at nine institutions around the United States began a pivotal phase 2/3 trial that included 123 pediatric patients who were randomized 1:10 to two arms. One received tozuleristide at a dose of $15\;\mathrm{mg}/m^{2}$ 1 to 36 h before surgery (n=112) and one received standard of care neurosurgery with no drug treatment (n=11). The study design focused on analysis of “equivocal tissue,” that is, normal tissue surrounding the tissue such as retraction artifact or gliosis that should be preserved. This is a particularly important analysis for the use of tumor paint in pediatrics as 85% of pediatric tumors present in the posterior fossa where it is highly valuable to visualize definitive margins. Enrollment for this trial has been completed including 63% males, diversity of race, and proportionate representation of four main pediatric CNS tumor types. There have been no drug-related serious adverse effects to date. Looking to the future, Dr. Lee shared updates regarding the blinded central fluorescence assessment required to complete the trial as well as device upgrades for technical modifications to the camera system (ClinicalTrials.gov, no. NCT02462629).

### 6.3 Clinical Experience Using ICG

Dr. Abdelhafeez H. Abdelhafeez from St. Jude Children’s Research Hospital presented on the use of ICG for pediatric tumor resection. Although adult clinical studies have indicated that ICG can improve tumor localization and resection, it is still unclear if it can be similarly applied to pediatric cancers. Dr. Abdelhafeez has conducted a retrospective study with St. Jude data from 2019 to 2020 to determine the feasibility of ICG-guided tumor resection in a broad range of childhood cancers including neuroblastoma, sarcomas, hepatic tumors, pulmonary metastases, and other rare pediatric tumors.^46^ A 1.5-mg/kg infusion of ICG 24 h prior to surgery is sufficient for detecting both primary tumors and metastatic deposits, indicating that the optimal dose for pediatric patients is much lower than the 3 to 5  mg/kg ICG typically used in adult clinical trials. In 65 procedures, there were four cases in which ICG localized tumors that were not identified by standard of care visualization and palpation (Table 6). Although NIR localization was successful in eight patients who underwent ICG-guided nephron-sparing surgery for pediatric renal tumors, Dr. Abdelhafeez’s team observed a pattern in which tumors were hypofluorescent and adjacent healthy kidney tissue was avidly fluorescent.^47^ Further work must be conducted to explore the feasibility of ICG-guided pediatric tumor resection, but these preliminary findings suggest that SWIG sensitivity and specificity differ depending on tumor histology and location (Tables 7 and 8).

**Table 6:** FDA-approved agents.

Presenter	Tracer	Target tumor	Mechanism of action
Walter Stummer, MD, PhD	5-ALA	Gliomas	Prodrug is metabolized in tumor cells to produce protoporphyrin-IX
Janos L. Tanyi MD, PhD	Pafolacianine (OTL-38)	Ovarian cancer	Targets cells with overexpression of FRα
Linda Martin, MD, MPH	Pafolacianine (OTL38)	Lung cancer	Targets cells with overexpression of FRα

**Table 7:** ICG-guided pediatric tumor resection by Abdalhafeez et al.^48^

Procedures n=65	True positives n=46	False positives n=3	True negatives n=10	False negatives n=6	Localization of tumors not detected by standard of care n=4
Thorax n=37	24	2	8	3	3
Abdomen n=19	13	1	2	3	0
Trunk and extremities n=9	9	0	0	0	1

**Table 8:** Outcomes of 2019-2020 ICG-guided pediatric tumor resection.

Sensitivity	88%
Specificity	77%
Positive predictive value	94%
Negative predictive value	63%
Accuracy	86%

ICG infusion may be used for lymph node sampling and staging of pediatric tumor. In eight patients with Wilms tumor, ICG was administered locally by IP injection for laparoscopic nephrectomy and by peri-hilar (PH) injection for open nephrectomy.^49^ Since the benefits of fluorescence-guided lymphatic mapping is still unclear, Dr. Abdelhafeez’s group will soon launch a prospective trial for ICG-guided SLN mapping during retroperitoneal lymph node dissection. His team is currently involved in an ongoing prospective trial to explore the use of ICG for identifying neoplastic disease during pediatric tumor resection. They are assessing for ICG uptake, the effect of chemotherapy and RT on fluorescence, localization, synchronous lesions, and positive margins.

### 6.4 Preclinical Experience on Target Agents for Pediatric Solid Tumors

Dr. Marcus M. Malek from the Children’s Hospital of Pittsburgh discussed radiolabeled and fluorescently labeled anti-GD2 probes. As radiological instruments continue to improve, surgeons are tasked with removing smaller microdeposits of disease. Dr. Malek’s team has developed a tracer that targets GD2, a highly expressed antigen in common childhood cancers including high-risk neuroblastoma and osteosarcoma.^50^^,^^51^

Neuroblastoma is the most common extracranial pediatric solid tumor. There are ∼700 to 800 new diagnoses each year in the United States, 70% of which have metastatic disease.^52^ Gross total resection is the surgical goal, but complications and inadequate surgery rate can be as high as 30% to 50%. IMI may reduce this rate by facilitating the detection of synchronous lesions, margin evaluation, and localization, which can limit the damage inflicted on vital neurovascular structures that are frequently encased by this tumor. Dr. Malek’s group is currently evaluating the use of dinutuximab (an FDA-approved anti-GD2 antibody) radiolabeled with indium-111 and conjugated to IRDye800CW for NIR surgical navigation. Their highly specific antibody-based tracer relies on gamma detection for improved localization and NIR fluorescence for lesion confirmation and margin evaluation.^53^

Dr. Malek’s group has tested the tracer on an orthotopic model of neuroblastoma in athymic, nude mice. 96 h after tail vein injection of In111-DTPA-diutuximab-IRDye800CW, mouse gamma biodistribution was performed. Tracer specificity was validated by increased uptake in tumors and reduced uptake in antibody blocking groups. Mice were further assessed with intraoperative systems including gamma detection probe (Mammotome, Cincinnati, Ohio) and with an NIR camera (Stryker, Kalamazoo, Michigan) both of which detected robust and tumor-specific tracer uptake. A processed tissue model was also utilized to assess the effect of tumor depth on signal detection. While fluorescent signal was significantly limited by depth within tissue, gamma signal could be detected from as deep as 5 cm within tissue. The next steps will be to optimize the time from injection to surgery, tracer dose, and molar activity of In111. In the near future, Dr. Malek will test signal detection limitations in tumors embedded in porcine organs as well as gelatin phantoms with autofluorescing or photon-absorbing background agents, and other GD2-positive tumors, such as melanoma, Ewing sarcoma, gliomas, and osteosarcoma.

## 7 Normal Tissue Dyes

### 7.1 Summary of Main Points

• Bevonescein
  • Bevonescein is a fluorescent dye first identified via phage display against human nerves.
  • Nerve imaging, assisted with bevonescein demonstrated higher visualization compared to white light.
• LGW16-03
  • Oxazine 4 is a nerve-specific fluorophore with an emission wavelength falls in the red-light spectrum.
  • LGW16-03 is a nerve-specific fluorophores that emit closer to 700 and 800 nm, which the use of current imaging system.
• IS-001
  • IS-001, a cyanine ureter probe, binds to urine proteins, and is renally metabolized.
  • Using IS-001 intraoperatively has allowed superior identification and delineation of the ureter in NIR Firefly images (Table 9).

**Table 9:** Normal tissue dyes.

Presenter	Tracer	Tissue type
Ryan Orosco, MD	Bevonescein (ALM-488)	Nerve
Summer L. Gibbs, PhD	LGW16-03	Nerve
Richard Farnam, MD	IS-001	Ureter

### 7.2 Nerve-Specific Dyes: Normal Tissue Identification

Bevonescein, a small protein related to fluorescein, is an investigational fluorescent dye first identified via phage display against human nerves. It has been found to not cross the BBB and has no biological effect upon binding. Dr. Ryan K. Orosco, MD, associate professor of otolaryngology at the University of New Mexico, described the first-in-human clinical trial (ClinicalTrials.gov, no. NCT04420689) of ALM-488 (Bevonescein) for intraoperative fluorescence nerve visualization.^54^

The objectives of this trial included understanding the safety of IV administration of this agent, characterizing the pharmacokinetics, dose, and timing, and appreciating the surgical impact of ALM-488 in terms of contrast enhancement, nerve length measurements, and branching delineation. Cases of neck dissection, parotidectomy, and thyroidectomy were included. The first component of the study involved a dose escalation schema starting at 100 mg of agent to 1000 mg of agent infused over 30 min assuming no dose limiting toxicities or adverse events. Once a safe dose with acceptable fluorescence was established, the second component of the study investigated the optimal dose timing schedule, that is, either 1 to 3 h or 2 to 5 h before surgery.

Twenty-seven patients were recruited between three sites with no serious adverse events related to the drug recorded. Surgeries were conducted per standard of care, with intermittent pausing to capture fluorescence images with the Zeiss Tivato camera system (Carl Zeiss Meditec AG, Jena, Germany). Urine and serum were collected for pharmacokinetic analysis, demonstrating a serum half-life of 30 min. Notably, the surgeons were able to observe fluorescence contrast up to 6 to 7 h after infusion at a dose of 500 mg. A quantitative analysis of SBR assessed by an independent core lab determined that 84% of paired nerve images at 500 mg dosage demonstrated higher contrast with bevonescein as compared to white light. In addition, 38% of nerves demonstrated an increased length of visualization using this agent. A visualization scoring system was provided to surgeons to quantify their evaluation of the contrast enhancement, length measurement, and branching delineation using the dye, which found a statistically significant improvement in each of these qualities. With these results, Dr. Orosco and his colleagues at four additional institutions are organizing a phase 3 trial with a target accrual of 200 patients.

### 7.3 Near-Infrared Nerve-Specific Probes for Intraoperative Nerve Visualization

Dr. Summer L. Gibbs from the Knight Cancer Institute at Oregon Health and Science University presented the use of NIR contrast agents for nerve imaging. Iatrogenic nerve damage is an important challenge with many surgical procedures. For example, head and neck surgeons often operate in close proximity to the facial nerve and vagus nerves. Similarly, nerve-sparing prostatectomy is dependent on identification of the neurovascular bundle. Specific nerve highlighting could potentially allow for better identification and visualization of nerves intraoperatively and reduce the chance of iatrogenic injury. One major consideration for nerve imaging is ensuring that the agent is nerve specific, to reduce the amount of imaging signal from fat, connective tissue, and glandular tissue. Oxazine 4 has been a promising nerve-specific fluorophore, but its emission wavelength falls in the red-light spectrum. Dr. Gibbs’ group has been working to chemically modify oxazine 4 to maintain its nerve specificity while shifting its emission into the NIR (650 to 900 nm) spectrum for surgical application. By adjusting the conjugation of agents such as oxazines 1 and 4, Dr. Gibbs’ group has been able to create nerve-specific fluorophores that emit closer to 700 and 800 nm, which allow these agents to remain compatible with current clinical fluorescence imaging systems.^55^

Currently, Dr. Gibbs’ group is moving one of their lead 700-nm nerve probes, LGW16-03, toward clinical translation. The group has studied LGW16-03 extensively in rats and mice, and the probe has shown a promising SBR when highlighting human nerves in patients who have undergone lower limb amputations in collaboration with Dr. Eric Henderson. LGW16-03 can also be potentially used with two-color fluorescence-guided surgery, to better identify and spare nerves that are near tumor tissue. In the CNS, the group has been able to visualize cranial nerves and white matter tracts using their NIR nerve imaging agents. They have also been able to perform functional fluorescence imaging to examine nerve health and assess the extent of prior nerve injury or detect iatrogenic nerve injury. The preclinical pharmacology and toxicology studies have been performed for LGW16-03, and the group is currently moving toward IND-enabling studies and clinical translation in collaboration with Dr. Eric Henderson at Dartmouth.

### 7.4 Ureter Dyes During Urologic Cancer Surgery: Normal Tissue Identification

Dr. Richard W. Farnam from Las Palmas Del Sol Healthcare presented a phase 2 multicenter study on the applications of IMI for urologic cancer. Minimally invasive procedures such as a laparoscopic hysterectomy are used to treat urologic disease but are associated with ureteral injury, particularly at the pelvic brim (PB) and cardinal ligament.^48^ IMI allowed for more distinct localization and depth perception, which can help prevent ureteral injury. Because ICG is hepatically metabolized, it cannot be used for intraoperative ureter visualization. Dr. Farnam’s group has developed IS-001, a cyanine ureter probe that is intravenously administered via a slow-bolus injection and is renally excreted. Its excitation (peak∼780  nm) and emission (peak∼815  nm) are similar to ICG, making it compatible with the clinically available Intuitive daVinci Robotic System and Firefly imaging mode.

After confirmation of clinical safety in the phase 1 data, the study moved to phase 2 and enrolled 94 subjects to SBR and efficacy.^56^ Dr. Farnam’s team has determined that signal can be detected through 0.5 to 1 cm of tissue and that the optimal imaging window is 30 to 45 min after infusion of 22 mg IS-001. SBRs were >2.0 at both the PB and uterine arteries (UA), indicating enhanced ureter visualization. Intraoperative assessment by the surgeon also suggested that NIR imaging significantly improved localization compared with white light. Ureter identification and delineation efficacies were assessed by 10 independent, blinded readers who were tasked with labeling the ureter on ∼300 randomized images in four different conditions: anatomical location (PB or UA), time-point, white light, or Firefly imaging, and presence or absence of ureter in the surgical field of view. At both anatomical locations, readers had superior identification and delineation in NIR Firefly images. By 60 min postinfusion, the fluorescent signal had attenuated. There were no clinically relevant adverse events. These results are promising, and a phase 3 multicenter study is in the pipeline to further evaluate the use of IS-001 in urologic cancer resection.

## 8 Bench to Bedside Tips

### 8.1 Choline Kinase Dyes

Dr. Edward J. (Jim) Delikatny, PhD, professor of radiology at the University of Pennsylvania reports recent developments of a choline kinase-targeted NIR dye for the imaging of lung cancers. Choline kinase is an enzyme that is critical in the biosynthesis of phosphatidylcholine which makes up roughly 50% of cell membranes. It is upregulated in 59% of nonsmall-cell lung cancer (NSCLC).^57^

Choline kinases inhibitors typically include bisymmetric aromatic rings containing a positively charged nitrogen group connected by a linker moiety. Delikatny’s group therefore used a similarly structured bis-indolium dye which is the backbone of cyanine dyes to synthesize a NIR dye JAS239 that would competitively inhibit choline kinase.^58^^,^^59^ This dye was validated in a panel of human breast cancer cell lines as well as mice bearing orthotopic MCF7 breast xenografts. At this point, Dr. Delikatny collaborated with thoracic surgery colleagues to target NSCLC which is the leading cause of lung cancers and has up to 40% recurrence at the primary site after surgical resection. To begin the translation from dye candidate to viable diagnostic tool, his group screened murine NSCLC cell lines for elevated choline kinase expression, identifying the KLN205 tumor line as a strong model. It metastasizes from a flank tumor to the lungs in roughly 20 days. Thereafter, they dosed JAS239 in this orthotopic murine model to confirm via biochemical and histologic analysis that JAS239 was nontoxic in mice.^58^

Clinical trials using companion dogs were initiated as companion canine models provide similar environmental conditions, cancer development, and optimal imaging techniques to humans. These dogs would receive JAS239 4 to 24 h before surgery during which the wound bed was examined for tumor margins, lymph nodes, and micrometastases and back-table analysis was then correlated with histology and IHC. Target validation was first completed in five canines with lung adenocarcinomas to confirm the increased choline kinase expression in the lung tumor as compared with benign lung. Toxicology studies were then completed in canines to ensure that JAS239 was nontoxic in normal canines. Therefore, the clinical trial was completed with 12 canines of mixed breed, age, weight, tumor size and location, and sex at various doses of JAS239 ranging from 0.25 to 1  mg/kg. Analyzing canine imaging by dose reveals the optimal TBR of 6 at 0.5  mg/kg, which is confirmed by back table imaging. Finally, recent data confirm the utility of JAS239 in delineating tumor from necrotic regions, detecting surgical margins, and identifying lymph node involvement. Moving forward, Dr. Delikatny’s group plans to complete the canine clinical trial (n=30) to further optimize dose and timing as well as technical components, before translating to human clinical trials.

### 8.2 pH-Sensitive Dyes: Baran Sumer, MD

Biomarker expression on tumor cells can be variable and may not be universal within even a single cancer type. As such, developing a universal imaging probe with high specificity and sensitivity presents unique challenges for cancer imaging. To do so, Dr. Baran Sumer, MD, Chief of Head and Neck Oncology at UT Southwestern Medical Center, in conjunction with Jinming Gai, PhD, professor of pharmacology, have developed an imaging drug that targets the uniquely acidic tumor microenvironment as the tumor pH has been found to be roughly 6.8 or lower across a wide range of tumor types, as compared with blood pH of 7.4.^60^ This drug has been previously described and is known as pegsitacianine.

Initial phase 1 and phase 2 trials were completed in Europe whereby a pH-nanoprobe was given to 17 patients intravenously 24 h before surgery at a dose of 1  mg/kg. It was able to detect fluorescence in a variety of different cancers including head and neck cancers with an estimated PPV of roughly 70%.^33^ This led to a phase 2 head and neck cancer clinical trial completed at the University of Pennsylvania in 14 patients, which demonstrated 0/14 positive margins, which is lower than previously reported values around 10%, indicating clear demarcation (i.e., no overlap) as well as a PPV of 83%, sensitivity of 100%, and specificity of 86%. To improve the specificity and minimize the background signal, Dr. Sumer’s group determined that using a polymer micelle that would degrade at a lower pH, perhaps as low as 5.3, would be a suitable solution.^61^^,^^62^ This led to the phase 2 clinical trial now organized to identify unknown primary cancers of the head and neck using now-improved pH-nanoprobe pegsitacianine. These tumors are uniquely posed to benefit from fluorescence-guided resection as many of these patients have large portions of their larynx and pharynx irradiated with high doses in treatment, leading to dysphagia. The phase 2 design is a nonrandomized, open-label, single-center safety, and imaging feasibility study of pegsitacianine. The standard primary endpoints are sensitivity, specificity, PPV, and proportion of patients with an occult disease detected or a positive margin missed by standard of care. Exploratory endpoints include TBR, number of previously unidentified margins detected, and reresection rate, among others.

### 8.3 NIH Perspective of Clinical Trials in Intraoperative Molecular Imaging

Dr. Lalitha K. Shankar from the National Cancer Institute (NCI) explained the different NCI funding opportunities for optical imaging clinical trials. The NCI Division of Cancer Treatment and Diagnosis supports phase 1 and early phase 2 trials through its Experimental Therapeutic Clinical Trial Network (ETCTN) and phase 2 and 3 trials through its National Clinical Trials Network (NCTN). Table 10 lists funding opportunities available. Dr. Shankar also introduced the NCI Experimental Therapeutics Program (NExT), which is a competitive contract program that provides access to NCI discovery and development resources for high-risk preliminary projects.

**Table 10:** NIH funding opportunities for IMI clinical trials.

Funding with ETCTN and NCTN	FY23 SBIR contract solicitation of interest
PAR-21-033: National Cancer Institute’s Investigator-Initiated Early Phase Clinical Trials for Cancer Treatment and Diagnosis (R01 clinical trial required).	Topic 452: Translation of novel cancer-specific imaging agents and techniques to mediate successful image-guided cancer interventions.
NOT-DE-21-D10 Notice of Special Interest (NOSI): precision imaging of oral lesions	Topic 454: Software to evaluate artificial intelligence/machine learning medical devices in oncology settings
PAR-22-071: toward translation of nanotechnology cancer interventions (TTNCI) (R01 clinical trial notallowed)	PHS 2022-2 Omnibus Solicitation of the NIH, CDC, and FDA for Small Business Innovation Research Grant Applications (Parent SBIR [R43/R44] clinical trial required)
PAR-22-091: Exploratory/Developmental Bioengineering Research Grants (EBRG) (R21 clinical trial optional)	—
PAR-21-206: Academic-Industrial Partnerships for Translation of Technologies for Diagnoses and Treatment (R01 clinical trial optional)	—

## 9 Alternative Imaging Strategies

### 9.1 Summary of Main Points

• Fluorescence lifetime imaging
  • Fluoresce lifetime is the decay map of a molecule that is excited by a laser pulse and can then be built by graphing the exponential decay against time.
  • The lifetime of the tumor is distinct from normal tissue with a statistically significant lifetime separation.
• Label-free fluorescence lifetime imaging
  • Label-free fluorescence lifetime imaging (FLIM) leverages the autofluorescence properties of structural proteins, metabolic cofactors (e.g., NADH, FAD), lipids, lipoproteins, and other molecular complexes to identify tumor tissue without the administration of exogenous contrast agents.
  • FLIM can provide optical contrast between areas of high and low tumor density and help identify several tumor subtypes.
• Photoacoustic imaging
  • The photoacoustic effect takes advantage of the nonradiative relaxation of photons, releasing energy as heat that can be transferred to sound waves by thermal expansion.
  • Sound has the advantage of experiencing much weaker scattering in biological tissue when compared with traditional fluorescence photons, making it suitable for high-resolution imaging in deep tissue.
• Multispectral camera imaging
  • Current NIR imaging instruments employ one to two fluorescent channels and are dye specific.
  • A multispectral camera uses vertically stacked photodetectors at various depths to detect different emission wavelengths and can differentiate fluorophores within the 700 nm and within the 800 nm wavelengths.

### 9.2 Fluorescence Lifetime Imaging with Dyes

Dr. Kumar from Massachusetts General Hospital, Harvard Medical School introduced a different method to quantify fluorescence than fluorescence intensity. He explained that fluorescence intensity has proven to be fast, portable, and easy to use.^63^ However, there are some disadvantages: it cannot always separate tumor from background, it depends on the imaging system being used. It can be affected by external ambient light and has a difficulty of multiplexing (detecting different dyes at the same time).

While the fluorescence spectrum is widely utilized, fluorescence lifetime is another area of quantifying contrast. It is influenced by the average time a molecule spends in an excited state (nanoseconds for infrared light). Once a molecule is excited by a laser pulse, a decay map can then be built by graphing the exponential decay against time (in seconds). Each pixel is fit for its corresponding exponential decay value over time to generate a lifetime map.

The advantage of this is that unlike MFI, which is unitless, this can be measured in nanoseconds. It has also been shown to be more sensitive than MFI and usable in the setting of multiple dyes (multiplexed imaging).^64^^,^^65^

Dr. Kumar’s early work was mainly focused on tomographic applications of lifetime in animals with applications including cardiac imaging with activatable dyes. In early 2016, injecting ICG in a mouse and imaging a day later showed that the lifetime of the tumor was distinct from normal tissue with a statistically significant lifetime separation. In comparing ICG fluorescence intensity to lifetime, lifetime had a superior ability to discern SBR compared with using fluorescence alone.^66^

Upon examining EGFR-targeted probes in preclinical models, the same phenomenon was observed. Lifetime was enhanced in tumors with a superior performance accuracy in comparison to intensity.^60^ Traditional measurement of fluoresce intensity of these specimens showed that fluorescence was detected outside the tumor margins. However, the lifetime map was more restricted to the tumor and had less background.^67^ Upon microscopic evaluation, there was a strong correlation between EGFR expression seen on IHC and the lifetime of anti-EGFR-conjugated IRDye800CW. To further corroborate the localization and discerning capacity of lifetime, Dr. Kumar presented a liver cancer specimen from a cancer resection done by Dr. Kenneth Tabe at MGH. The tumor stained with ICG shows peripheral enhancement and no fluorescence within the tumor. A comparative evaluation using lifetime showed that the entire tumor actually fluoresced, especially areas that did not fluoresce on ICG alone. Dr. Kumar also showed examples from various types of cancer tissues and showed that the mean lifetime was significantly longer. This could allow for a fluorescence lifetime threshold that can be applied across multiple tumor types.

Dr. Kumar showed the application of this technology can be expanded to lymph node evaluation and multiplexing. In oral cancer surgery performed by Dr. Allen Feng at Massachusetts Eye and Ear, lymph nodes were examined using tumor lifetime. The negative lymph node had shorter lifetimes and correlated well with histology. In brain tumor imaging of specimens sent from Dr. John Lee at the University of Pennsylvania, tumor lifetime not only allowed detection of tumor cells but also discerning blood vessels, part of its multiplexing capacity.

Dr. Kumar then explained that while work is still progressing in understanding the mechanisms behind lifetime increase in solid tumors, a tentative explanation for why lifetime differentiates malignant from benign cells has to do with dye endocytosis and higher lysosomal viscosity of cancer cells.

### 9.3 Label-Free Fluorescence Lifetime Imaging

Dr. Laura Marcu PhD, professor of biomedical engineering and neurological surgery at University of California–Davis described label-free optical technologies in the surgical workflow. Label-free FLIM leverages the autofluorescence properties of structural proteins, metabolic cofactors (e.g., NADH, FAD), lipids, lipoproteins, and other molecular complexes to identify tumor tissue without the administration of exogenous contrast agents.^68^ The major challenges preventing the application of FLIM in the operating room involve the complex and expensive instruments with long data acquisition times required for FLIM. As such, Dr. Marcu has developed practical point-scanning FLIM devices such as the pulse-sampling multispectral FLIM instrumentation. It records fluorescence decay using a tandem of four separate spectral channels that were tailored to molecular components in the tissue.^69^ When digitized, this compact apparatus allows for fast, accurate, and precise multispectral FLIM measurements. Importantly, this approach enables FLIM measurements under standard room-light illumination and allows the surgeon to easily navigate the complex geometry of resection cavities. This device is used in conjunction with a surgical microscope/endoscope and robotic platform to provide a real-time augmented FLIM image on the surgical field-of-view.

In addition, her group has developed FLIM fiber probes for different clinical applications, such as robotic surgery, brain biopsy guidance, transoral surgery, and neurosurgery. In transoral surgery, they are able to correlate FLIM data with histopathology results to develop classifiers based on fluorescence parameters that could directly augment tumor probability predictions.^70^ Furthermore, this technique was useful to correctly detect primary lesion in patients with p16+ head and neck squamous cell carcinoma of the unknown primary and demarcate entire benign tissue from oropharyngeal carcinoma.^71^ In neurosurgery, Alfonso-Garcia et al. demonstrated safe and real-time intraoperative FLIM acquisition which was correlated to histology and MRI to resolve distinct brain tissue types that were clinically relevant.^72^ Dr. Marcu also reported leverage of this technology to distinguish oligodendroglioma from astrocytoma indicating that label-free FLIM could be a biomarker for low-grade IDH-mutant tumors.^73^ Preliminary results suggested that FLIM data can provide optical contrast between areas of high and low tumor density thereby detecting the infiltrative edge of GBM in real-time. Dr. Marcu also describes quantitative assessment of PpiX fluorescence at room light using FLIM as an augmented visualization tool for real-time resection under standard surgical field illumination.^74^

### 9.4 Photoacoustic Imaging

Dr. Lei Li from Rice University presented the clinical applications of advances in photoacoustic imaging. Optical imaging allows for the characterization of the functional, metabolic, anatomical, histological, and molecular properties of tissue. Photoacoustic tomography has the advantage of imaging *in-vivo* organs up to 4 cm deep. The photoacoustic effect takes advantage of the nonradioactive relaxation of photons, releasing energy as heat that can be transferred to sound waves by thermal expansion.^75^ Sound has the advantage of experiencing much weaker scattering in biological tissue when compared with traditional fluorescence photons, making it suitable for high-resolution imaging in deep tissue. Taking advantage of this light-matter interaction, photoacoustic imaging has been able to use various endogenous chromophores and exogenous contrast agents, such as hemoglobin, melanin, lipids, DNA/RNA, organic dyes, nanoparticles, and fluorescence proteins. Another advantage of photoacoustic imaging is that its spatial resolution and penetration depths are scalable with the detection frequency. Photoacoustic CT works by shining a laser light onto a tissue, which raises the temperature of the tissue a bit and leads to volume expansion, generating acoustic waves. Acoustic sensors can then generate high-resolution images mapping the optical absorption inside deep tissue.

Dr. Li talked about whole-body photoacoustic CT at high spatiotemporal resolutions and its applications in rodents and humans.^76^ Using single-impulse panoramic photoacoustic CT, a resolution of 100  μm across the whole body of an animal can be reached with a data acquisition time of 50  μs at a framerate of 50 Hz. Photoacoustic can allow for real-time imaging of the abdomen, liver, and brain of rodents. It can also perform *in vivo* functional imaging of the whole brain of rats, allowing them to map out the functional connectivity of the brain and image the detailed vessel networks within the brain via optical imaging techniques. Photoacoustic have also been applied to image melanoma cells in small animal models, using endogenous melanin as the contrast agent (absorption near 690 nm).^4^ Dr. Li’s team has started moving toward clinical translation by creating a panoramic photoacoustic CT imaging system for human breast tumor diagnosis and assessing blood vessel density. Functional photoacoustic imaging can also be applied to the human brain. It has a higher temporal resolution when compared with MRI and provides a potentially cheaper alternative to magnetic field-based imaging.

### 9.5 Multispectral Camera Imaging

Dr. Victor Gruev from the University of Illinois at Urbana-Champaign discussed the applications of bioinspired imaging devices for NIR surgical navigation. Current NIR imaging instruments employ one to two fluorescent channels and are dye specific. However, it can be costly and inefficient to purchase a new imaging device for each tracer. This also limits the simultaneous use of multiple fluorophores intraoperatively, which is an approach that may improve sensitivity and specificity compared with a single tracer infusion. To resolve this issue, Dr. Gruev’s team is developing a camera inspired by the mantis shrimp’s optical system. The imaging device uses vertically stacked photodetectors at various depths to detect different emission wavelengths.^77^ It is highly specific and can differentiate fluorophores within the 700 nm and within the 800 nm wavelengths. Furthermore, it is sensitive to small spectral changes and can distinguish between ICG diluted in water and ICG diluted in serum, which are only spectrally separated by 8 nm.

Dual-tracer strategies have shown promising results for improved sensitivity and specificity. Dr. Gruev’s group has tested this approach in a murine model of human prostate cancer. EGF conjugated to IRDye680RD and 2-DG conjugated to IRDye800CW were intravenously administered. After 24 h, each tracer fluorescently labeled tumor tissue that would have otherwise gone undetected by the other probe, suggesting that combination tracers have a complementary effect. The imaging system is also able to distinguish multiple fluorophores in human patients. Dr. Gruev’s clinical study has shown that local administration of methylene blue and ICG assists with SLN mapping in 25 patients with breast cancer. Future studies will further evaluate the benefits of multiple fluorophores for *in vivo* surgical navigation.

Another limitation to current imaging technology is the endoscope itself. Because NIR fluorescence and color in the visible light spectrum are focused at different points, imaging both views simultaneously with a standard endoscope requires multiple cameras and reduces resolution.^78^ Dr. Gruev’s team has developed a single-camera NIR endoscope that successfully focuses NIR and color images at the same plane. The endoscope is compatible with their bioinspired single-camera system, which improves resolution compared with standard endoscopes (Karl Storz, Tuttlingen, Germany) and eliminates the need for multiple NIR cameras.

The multispectral imaging system has demonstrated the potential to differentiate tracers within the 600- to 800-nm emission peak range and determine the exact ratio of multiple fluorophores within fluorescent tissue. Currently, Dr. Gruev’s group is testing an ultrasensitive NIR imaging sensor (<1  ms integration time) in an ongoing clinical trial for a cathepsin-activated NIR tracer (VGT-309) in pulmonary cancer. The goal of this camera is to capture weak fluorescence observed in small metastases and deep tumors that may be undetected by other conventional imaging devices.

### 9.6 Unmet Needs

There are various applications of IMI: identifying margins, identifying synchronous lesions, localizing occult cancers, identifying normal structures, performing complete cytoreductive surgery, and even identifying select positive lymph nodes. However, there remains some scientific challenges and unmet needs.

To begin, Dr. Singhal explained that background noise remains a common problem. Part of the reason is secondary to autofluorescence and background binding. The second is lymph node imaging. Although there has been some discussion about it in head and neck surgery, it remains an unmet challenge. SLNs are widely used but carry a heavy burden such as lymphedema. Up to 10% of patients developing upper extremity lymphedema even with SLD biopsies.^79^ Furthermore, micrometastases imaging is an area that we have not been able to address fully yet. The tracers are available but the cameras we currently have might not be sensitive enough to detect micrometastases. The challenge is to maintain our clinically useful field of view in the OR with adequate resolution to identify micrometastatic disease. Its importance is twofold. On the one hand, knowing that there is remaining microscopic metastasis can help guide the need for postoperative chemotherapy or other treatments. Also, leaving cells behind in a pool of blood or fluid that have no ability to adhere to the resection bed and proliferate is different from leaving tumor cells with the ability to multiply and cause a recurrence.

Another issue is depth of penetration. NIR allows for better depth penetration than visible wavelengths, but NIRII imaging is another unmet need. This second NIR window (1000 to 1700 nm) has maximum permission exposure and penetration depth over NIR-I phototherapy (650 to 950 nm) but has not been fully used in IMI at this time.^80^ On another note, current applications have not addressed renal cell, bladder, pancreatic, and endometrial cancer, all of which could benefit from this technology. Finally, endoscopic approaches remain a challenge. There are no commonly available flexible endoscopic approaches that utilize fluorescent imaging.

The future is phase 4 trials and that entails several steps from a regulatory standpoint. The FDA requirements for IMI will fall under diagnostic requirement. These are going to be more strict than therapeutic as these tests are diagnostic. Another area worth further investigation is adoption of these companies by hospital systems. We currently have a wide range of dyes and multiple cameras that can detect multiple dyes. Will hospitals buy additional cameras or favor using dyes that work on already existing cameras that they have? Another is addressing reimbursement. There are no reimbursement strategies currently in place for IMI. In light of that, there is value in identifying who would benefit most from IMI. Ideally, a predictive tool to identify who would benefit from undergoing IMI would be helpful in guiding patient selection.

Another important tool in encouraging adoption and proper utilization of IMI technologies is education particularly after a drug undergoes a successful phase 3 clinical trial. Although a lot of the technicalities of using this technology are rather straightforward, there is an element of learning how to discern false positives and interpreting images. In other words, how would surgeons become adept at discerning TBR ratios adequately intraoperatively. The consensus was organizing courses afterward to disseminate educational material. From the surgeons’ experience, maintaining engaging educational activities as opposed to mere distribution of educational materials (flyers or videos) yields better results in disseminating information and ensuring safe and proper adoption of the technology. Dr. Singhal concluded with an optimistic statement that the negative predictive value of this technology is very impressive. He explained that if after the conclusion of surgery there is remaining fluorescence, it could either be a false positive or a true positive. Meanwhile, if after cancer surgery no fluorescence is detected, then it is likely a true negative and this is reassuring.
